# *Medicago truncatula SOC1* Genes Are Up-regulated by Environmental Cues That Promote Flowering

**DOI:** 10.3389/fpls.2018.00496

**Published:** 2018-04-27

**Authors:** Jared B. Fudge, Robyn H. Lee, Rebecca E. Laurie, Kirankumar S. Mysore, Jiangqi Wen, James L. Weller, Richard C. Macknight

**Affiliations:** ^1^Department of Biochemistry, University of Otago, Dunedin, New Zealand; ^2^Plant Biology Division, Samuel Roberts Noble Foundation, Ardmore, OK, United States; ^3^School of Biological Sciences, University of Tasmania, Hobart, TAS, Australia; ^4^New Zealand Institute for Plant and Food Research Ltd., University of Otago, Dunedin, New Zealand

**Keywords:** flowering time, *Medicago*, photoperiod, vernalization, legume, genome evolution

## Abstract

Like *Arabidopsis thaliana*, the flowering of the legume *Medicago truncatula* is promoted by long day (LD) photoperiod and vernalization. However, there are differences in the molecular mechanisms involved, with orthologs of two key *Arabidopsis thaliana* regulators, *FLOWERING LOCUS C* (*FLC*) and *CONSTANS* (*CO*), being absent or not having a role in flowering time function in *Medicago*. In *Arabidopsis*, the MADS-box transcription factor gene, *SUPPRESSOR OF OVEREXPRESSION OF CONSTANS 1* (*AtSOC1*), plays a key role in integrating the photoperiodic and vernalization pathways. In this study, we set out to investigate whether the *Medicago SOC1* genes play a role in regulating flowering time. Three *Medicago SOC1* genes were identified and characterized (*MtSOC1a–MtSOC1c*). All three *MtSOC1* genes, when heterologously expressed, were able to promote earlier flowering of the late-flowering *Arabidopsis soc1-2* mutant. The three *MtSOC1* genes have different patterns of expression. However, consistent with a potential role in flowering time regulation, all three *MtSOC1* genes are expressed in the shoot apex and are up-regulated in the shoot apex of plants in response to LD photoperiods and vernalization. The up-regulation of *MtSOC1* genes was reduced in *Medicago fta1-1* mutants, indicating that they are downstream of *MtFTa1.* Insertion mutant alleles of *Medicago soc1b* do not flower late, suggestive of functional redundancy among *Medicago SOC1* genes in promoting flowering.

## Introduction

In annual plants, the transition from vegetative growth to flowering, termed floral induction, is regulated by environmental and endogenous cues to promote flowering in spring time (Srikanth and Schmid, 2011; Letswaart et al., 2012; Romera-Branchat et al., 2014). In the Brassica *Arabidopsis thaliana*, the key environmental cues which promote flowering are exposure to a prolonged period of cold (vernalization), followed by long day (LD) photoperiods. Endogenous signals such as carbohydrate status, gibberellin metabolism, developmental stage, and the autonomous floral promotion pathway also interact to promote flowering (Andres and Coupland, 2012; McClung et al., 2016; Cheng et al., 2017). In *Arabidopsis*, floral induction is repressed in non-inductive conditions by the MADS-box transcription factors *FLOWERING LOCUS C* (*FLC*) and *SHORT VEGETATIVE PHASE* (*SVP*) (Andres and Coupland, 2012). These floral repressors bind to regulatory elements of *FLOWERING LOCUS T* (*FT*) and *SUPPRESSOR OF OVEREXPRESSION OF CONSTANS 1* (*SOC1*) and other genes, such as those involved in gibberellin metabolism (Hepworth et al., 2002; Searle et al., 2006; Tao et al., 2012; Mateos et al., 2015). Vernalization downregulates *FLC* expression by epigenetic and non-coding RNA-based mechanisms (Whittaker and Dean, 2017). *SVP* represses flowering under short day (SD) photoperiods but has reduced expression under LDs in the inflorescence meristem and under high ambient temperatures (Hartmann et al., 2000; Lee et al., 2007). Flowering is then promoted in LDs by the stabilization of the zinc finger transcription factor CONSTANS (CO) protein. *CO* is expressed in leaf phloem companion cells and activates the expression of the *FT* gene, which encodes a mobile florigen signal protein (Kardailsky et al., 1999; Wigge et al., 2005; Corbesier et al., 2007; Notaguchi et al., 2008). Studies in rice have shown that FT protein moves to the shoot apical meristem (SAM), whereupon it interacts with bZIP transcription factor *FLOWERING D* (*FD*) via a 14-3-3 protein-mediated complex (Abe et al., 2005; Taoka et al., 2011) to activate expression of the MADS box genes *SOC1* and floral meristem identity (FMI) gene *APETALA 1* (*AP1*) (Abe et al., 2005; Wigge et al., 2005; Yoo et al., 2005), thereby inducing the floral transition.

In *Arabidopsis*, *SOC1* is a ‘floral integrator’ gene that perceives inputs from the vernalization, LD photoperiodic, and gibberellin pathways to promote flowering (Lee et al., 2000; Onouchi et al., 2000; Samach et al., 2000; Hepworth et al., 2002; Moon et al., 2003; Yoo et al., 2005; Helliwell et al., 2006; Searle et al., 2006; Sheldon et al., 2006; Jung J.H. et al., 2012; Hou et al., 2014). *SOC1* is also regulated post-transcriptionally (Song et al., 2009; Kim et al., 2013). Upon activation at the shoot apex, the SOC1 protein binds its own regulatory sequences (Immink et al., 2012; Tao et al., 2012) and interacts with AGAMOUS-LIKE 24 (AGL24), for translocation to the nucleus, thereby providing positive feedback from inductive floral cues (Lee et al., 2008; Liu et al., 2008; Torti and Fornara, 2012). Proper integration of inductive floral cues by *SOC1* is, therefore, an important step in regulating floral induction in *Arabidopsis*.

Despite strong conservation of flowering time genes between *Arabidopsis* and legume species, differences in the regulation and function of genes controlling flowering in legumes are becoming progressively more apparent (Weller and Ortega, 2015). *Medicago truncatula* (*Medicago*, *Mt*) and the garden pea (*Pisum sativum*, *Ps*), are vernalization-responsive, long-day annual legume species which are emerging models for flowering time studies in the agronomically important Fabaceae family (Clarkson and Russell, 1975; Laurie et al., 2011; Putterill et al., 2013; Weller and Ortega, 2015). The most striking difference is the absence of a clear *FLC* ortholog in legume species (Hecht et al., 2005). Expansions of flowering time genes are typical, such that *SVP*, *CO-LIKE (COL)*, *FT*, *SOC1*, and *FRUITFUL* (*FUL*) orthologs typically occur in multi-gene families in *Medicago*, garden pea, and soybean (*Glycine max*, *Gm*) (Hecht et al., 2005; Kim et al., 2012; Jung C.H. et al., 2012; Putterill et al., 2013). Yet, differences in gene function seem to have arisen – *MtSVP* genes do not delay flowering when over-expressed in transgenic *Medicago* (Jaudal et al., 2014), and *MtCOL* genes do not appear to regulate photoperiodic flowering (Wong et al., 2014). Insights have been gained into the function and regulation of garden pea and *Medicago FT* genes by inductive seasonal cues. PsFTa1/GIGAS is a LD photoperiod inducible and graft-transmissible mobile signal which promotes flowering in garden pea (Hecht et al., 2011). In *Medicago*, *MtFTa1* is the sole target of the vernalization pathway and is rapidly up-regulated in leaves in response to LD photoperiod (Laurie et al., 2011). Transgenic *Medicago* over-expressing *MtFTa1* flowers very early, while loss-of-function *fta1-1* mutants flower late and no longer respond to vernalization (Laurie et al., 2011). *Medicago spring* mutants flower early without vernalization and have elevated levels of *MtFTa1* when grown in LD photoperiods (Jaudal et al., 2013; Yeoh et al., 2013). However, it is unclear how vernalization or LD activates *Medicago FT* expression and how *FT* triggers flowering.

Here, we examined the role of three *Medicago SOC1* homologs (*MtSOC1a*, *MtSOC1b*, and *MtSOC1c*). Our study indicates that all three *MtSOC1*s are up-regulated by favorable seasonal cues in the shoot apex, via both *MtFTa1*-dependent and *MtFTa1*-independent pathways, and likely play a role in the regulation of *Medicago* flowering.

## Materials and Methods

### Sequence and Phylogenetic Analyses

*MtSOC1* cDNA sequences were obtained as described previously (Hecht et al., 2005). AtSOC1 was used as a query for a BLASTP search of GenBank. Predicted SOC1 amino acid sequences belonging to Rosid species were aligned using the MUSCLE algorithm (Edgar, 2004) in Geneious software version 10.0.2 (Biomatters). A neighbor-joining phylogenetic tree was constructed using aligned amino acid residues 61–170, which excluded the variable C-terminal domain and the conserved MADS DNA-binding domain.

### Plant Materials and Growth Conditions

*Medicago truncatula* wild-type accessions Jester and R108 were grown as previously described (Laurie et al., 2011). *fta1-1* (R108 background) was described by Laurie et al. (2011). Tnt1 retroelement insertions in the R108 background at the *MtSOC1b* locus (Medtr8g033250) were sourced from the Noble Foundation collection (Tadege et al., 2008). Primers for genotyping and quantitative real-time RT-PCR (qRT-PCR) are listed in Supplementary Table 1. Flowering time was scored by counting the number of nodes to the first flower on the main stem. *Arabidopsis thaliana soc1-2* (Columbia) mutants were described in Lee et al. (2000). *Arabidopsis* plants were grown and flowering time scored by counting the total number of rosette leaves at flowering (Laurie et al., 2011).

### Generation of Binary Constructs and *Arabidopsis* Transformation

The DNA was amplified by PCR using Platinum High Fidelity Taq DNA polymerase (Invitrogen) and cloned into binary expression constructs by Gateway^®^ LR recombination technology. Construct integrity was verified by Sanger sequencing and restriction digest. *Arabidopsis soc1-2* (Col) (Lee et al., 2000) was transformed in accordance with Narusaka et al. (2010) with *Agrobacterium tumefaciens* GV3101 harboring the constitutive expression (*CaMV 35S*) construct pB2GW7 (Karimi et al., 2002), incorporating the coding sequences of *AtSOC1*, *MtSOC1a*, *MtSOC1b*, and *MtSOC1c*. Transgenic plants were selected on soil by spraying with 0.02% BASTA (glufosinate).

### Quantitative Real-Time PCR (qRT-PCR)

Total RNA was isolated from *Medicago* and *Arabidopsis* and 1 μg used for cDNA synthesis, as described previously (Laurie et al., 2011). qRT-PCR was performed in 10 μL reactions using 3 μL of cDNA diluted 1/30, with Roche SYBR Green I master mix, in the Roche LightCycler 480 instrument. Multi-well plates were loaded using a CAS1200 PCR robot. Calculation of the relative gene expression levels was based on the ΔΔ*C*t method (Pfaffl, 2001). In *Medicago*, the data were normalized to the reference gene, *PROTODERMAL FACTOR 2* (*PDF2*) (Kakar et al., 2008). In *Arabidopsis*, the data were normalized to *ACTIN2* (Lee et al., 2013). See Supplementary Table 1 for qRT-PCR primer sequences.

### Statistical Analyses

Differences between means for treatments in gene expression data, and for flowering times of transgenic lines compared to the *Arabidopsis soc1-2* mutant, were assessed by one-way ANOVA at the 0.05 significance level, with corrections for multiple comparisons as stated, using GraphPad Prism version 7.0.

## Results

### An Ancient Genome Duplication Has Resulted in Two Distinct Classes of *SOC1* Genes in Legumes

**FIGURE 1:**
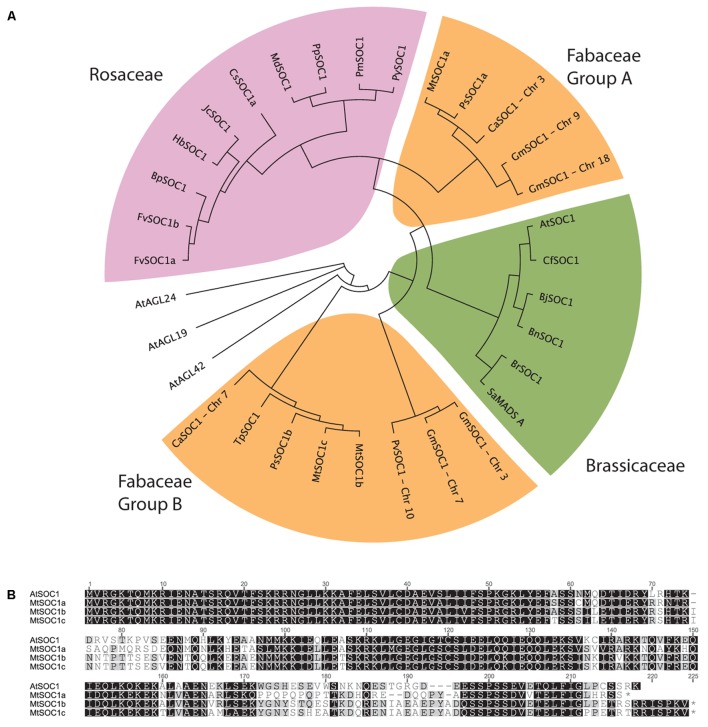
Phylogenetic relationships among SOC1 proteins. **(A)** Phylogeny of Rosid SOC1 proteins showing two distinct clades of Fabaceae SOC1, based upon a neighbor-joining tree from a MUSCLE alignment of residues 61–170, comprising a region spanning the Intervening and Keratin domains. Related *A. thaliana* AGAMOUS-LIKE sequences were used as outgroups. Species abbreviations: At: *Arabidopsis thaliana*, Bj: *Brassica juncea*, Bn: *Brassica napus*, Bp: *Betula platyphylla*, Br: *Brassica rapa*, Ca: *Cicer arietinum*, Cf: *Cardamine flexuosa*, Cs: *Citrus sinensis*, Fv: *Fragaria vesca*, Glyma: *Glycine max*, Hb: *Hevea brasiliensis*, Jc: *Jatropha curcas*, Md: *Malus x domestica*, Mt: *Medicago truncatula*, Phvul: *Phaseolus vulgaris*, Pm: *Prunus mume*, Pp: *Pyrus pyrifolia*, Py: *Prunus x yedoensis*, Ps: *Pisum sativum*, Sa: *Sinapis alba*, Tp: *Trifolium pratense*. **(B)** Alignment of full-length *Arabidopsis thaliana* (Col-0) and *Medicago* (R108 accession) SOC1.

The full-length AtSOC1 amino acid sequence was used as a BLASTP query to search for predicted SOC1 orthologs in GenBank. Sequences from 21 Rosid species were retrieved and aligned using the MUSCLE algorithm. A region 110 amino acids long corresponding to positions 61–170 (spanning the Intervening and Keratin domains, excluding the highly conserved MADS DNA-binding domain and highly variable C-terminal domain) was used to generate a neighbor-joining phylogenetic tree (**Figure 1A**). As a control, related AGL sequences (AGL19, AGL24, and AGL42) from *Arabidopsis* were included and did not cluster with any of the predicted SOC1 orthologs. Two distinct classes of SOC1 proteins were observed for Fabaceae members. We have named them Fabaceae Group A and B SOC1s (**Figure 1A**). *Medicago* has three *SOC1* genes, *MtSOC1a* (Medtr07g075870), *MtSOC1b* (Medtr08g033250), and *MtSOC1c* (Medtr08g033220) (Hecht et al., 2005). All three MtSOC1 (*c.v.* R108) genes encode proteins sharing 65–67% amino acid identity with AtSOC1 and were identical over the N-terminal MADS domain that is important for DNA-binding activity (**Figure 1B**). MtSOC1b and MtSOC1c proteins are highly similar to one another, sharing 93% identity (**Figure 1B**).

To investigate the evolution of the two groups of legume *SOC1* genes, we examined their genomic regions in diverse legume species. This revealed that a genomic region containing approximately 20 genes had been duplicated (Supplementary Figure 1). The synteny of this duplicated region has remained largely conserved. However, there are differences which can be used to determine which region likely contains the Group A or B *SOC1* genes. For example, the region containing *MtSOC1b* also includes a predicted deacetylase gene (Medtr08g033340) that is not present in the region with the *MtSOC1a* locus (a Group A *SOC1*). All the legume species we examined possessed this duplication, with the duplication existing in diverse taxonomic clades of papilionoid legumes; the phaseoloid or warm season legumes (such as soybean, common bean, cowpea, and pigeon pea), the galegoid or temperate legumes (*Medicago*, garden pea, and chickpea), and in lupin and peanut species, which belong to more distantly related clades (Genistoids and Dalbergioids, respectively). This indicates that the duplication likely occurred during the early evolution of the legume family, likely as a result of a whole genome duplication (WGD) event that occurred about 58 million years ago during the early evolution of the papilionoid legumes (Bertioli et al., 2009). Also in *M. truncatula*, there is a duplication of the Group B *SOC1* genes, resulting in *MtSOC1b* and *MtSOC1c* genes (**Figure 1A**, Supplementary Figure 1). This duplication is also present in *M. sativa* (*Ms*, alfalfa) (Supplementary Figure 2), but is absent in the other legumes, indicating that it occurred more recently.

### The *MtSOC1* Genes Can Partially Complement an *Arabidopsis soc1-2* Mutant

**FIGURE 2:**
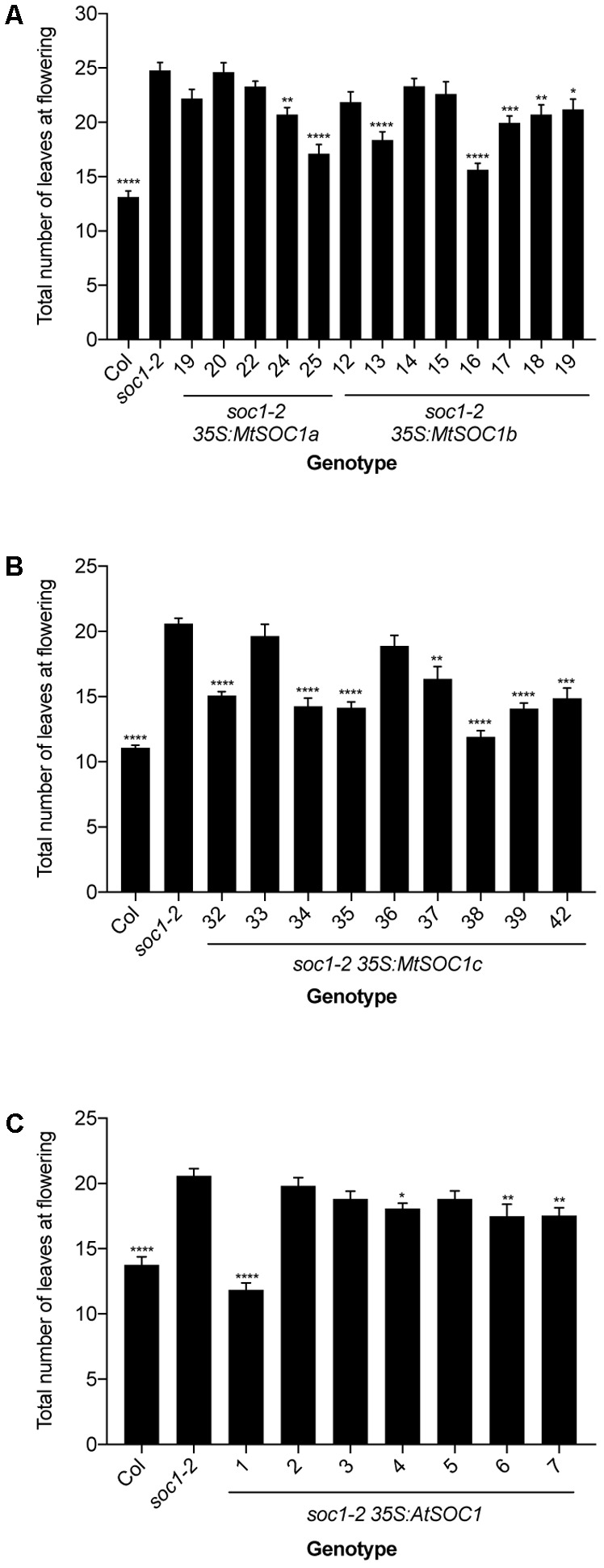
Complementation of late-flowering Arabidopsis *soc1-2* (Col) mutants by *Medicago SOC1* genes. Flowering time phenotypes of genetically independent transgenic plant lines ectopically expressing *SOC1* transgenes in the *soc1-2* mutant background. **(A)** Flowering time of homozygous T_3_ transgenic lines harboring *35S:MtSOC1a* or *35S:MtSOC1b* cassettes. **(B)** Flowering time of homozygous T_3_ transgenic lines harboring a *35S:MtSOC1c* cassette. **(C)** Flowering time of T_2_ transgenic lines harboring a *35S:AtSOC1* cassette. Data are mean ± SD of at least 12 plants grown under 16 h illumination at ∼120 μE.m^2^.s^-1^ light intensity at 22°C day time/18°C night time. Statistically significant differences between means of *soc1-2* versus each other genotype were determined by one-way ANOVA. Asterisks, where annotated, denote *P*-values. ^∗^*P* ≤ 0.05, ^∗∗^*P* ≤ 0.01, ^∗∗∗^*P* ≤ 0.001, ^∗∗∗∗^*P* ≤ 0.0001.

To investigate the function of the three *MtSOC1* genes, the ability of each gene to complement the *Arabidopsis* late-flowering *soc1-2* (Col) mutant (Lee et al., 2000) was examined. A series of transgenic plant lines expressing the coding sequence of each *MtSOC1* gene under the control of the constitutive *CaMV 35S* promoter were generated, in the *soc1-2* (Col) mutant background. For each *MtSOC1* gene, multiple transgenic lines were identified that promoted the flowering of *soc1-2* mutant to varying extents, as inferred by flowering times statistically significantly different to *soc1-2* (**Figures 2A,B**). As a control, the *Arabidopsis SOC1* gene was also re-introduced into the *soc1-2* mutant. Only one of seven lines (line 1) expressing *AtSOC1* completely rescued the *soc1-2* late-flowering mutant phenotype, meanwhile three lines partially rescued (lines 4, 6, and 7 flowered statistically significantly earlier than *soc1-2*) (**Figure 2C**). To investigate the possibility that transgene expression level contributed to the variation in flowering time observed, we isolated RNA from seedling tissues of additional transgenic lines containing *35S:MtSOC1* cassettes in the *soc1-2* background and examined transgene expression levels by qRT-PCR. Generally, the earlier flowering lines had higher levels of *SOC1* expression (Supplementary Figure 3). Overall, these results indicate that all three *MtSOC1* genes can promote flowering and are functionally equivalent to the *Arabidopsis SOC1* gene.

### The *MtSOC1* Genes Have Distinct Expression Profiles

**FIGURE 3:**
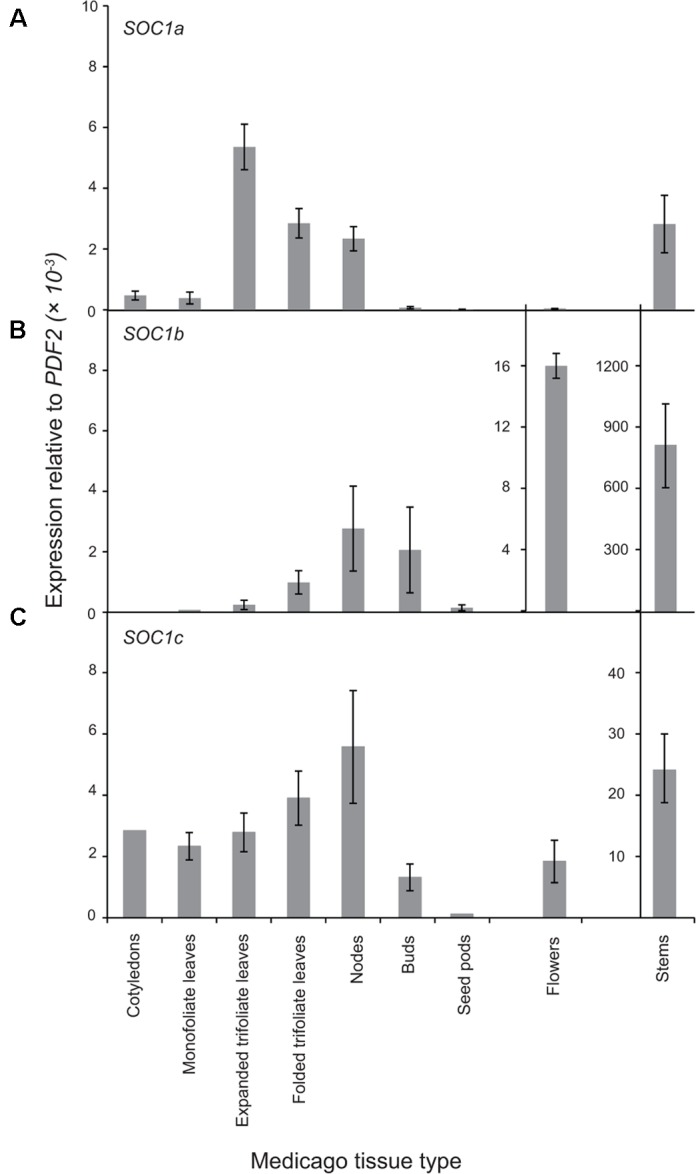
Expression pattern of *MtSOC1* genes. **(A)** *MtSOC1a*, **(B)** *MtSOC1b*, and **(C)** *MtSOC1c*. RNA was isolated from various tissues of WT *c.v*. Jester plants grown under inductive conditions. Vegetative tissues were harvested 15 days after sowing and reproductive tissues 35 days after sowing in soil. Data are the mean ± SE of three biological replicates and transcripts were normalized to *PDF2*. All tissues were harvested at ZT 2.

Next, the expression pattern of the three *MtSOC1* genes was analyzed by qRT-PCR using primers specific for each *MtSOC1* gene (Supplementary Figure 4). All three *MtSOC1* genes are expressed (**Figure 3**). *MtSOC1a* is expressed in vegetative tissues (leaves, stem, apical nodes), but is almost undetectable in reproductive tissues (floral buds, flowers, and seed pods) (**Figure 3A**). In contrast, *MtSOC1b* and *MtSOC1c* have a similar but not identical pattern of expression, and both are detected in vegetative tissues, in floral buds, and in flowers (**Figures 3B,C**). Consistent with a potential role in floral induction, all three *MtSOC1* genes are expressed under inductive environmental conditions in apical node tissue, containing the shoot apical meristem (SAM) (**Figure 3**). All three *MtSOC1* genes are also expressed in stem internode tissue, with *MtSOC1b* and *MtSOC1c* being expressed at the highest level in this tissue.

To determine whether the Group A and B *SOC1* genes also have distinct patterns of expression in other plants, we first examined available RNAseq data from *Medicago sativa* (The Alfalfa Gene Index and Expression Atlas Database^1^). This data also revealed that the three alfalfa *SOC1* genes are expressed at their highest levels in stem internodes. *MsSOC1a* was expressed at relatively low levels in leaves compared with the *MsSOC1b* and *MsSOC1c* genes. For all three *MsSOC1* genes, low or no expression was detected in developing flowers, nor root nodules, while modest expression was found in roots (Supplementary Figure 5A). Next, we examined the expression of the soybean *SOC1* genes in the RNA Seq Atlas database available at https://soybase.org/ (Severin et al., 2010). This revealed that *GmSOC1b* from chromosome 3 is only expressed at low levels in the tissues examined (young leaves, flowers, developing seeds, roots, and nodules), whereas the other *GmSOC1b* (from chromosome 7) and the Group A *GmSOC1*s from chromosomes 9 and 18 had somewhat similar patterns of expression, with low levels of expression in developing seeds, and modest expression in the other tissues (Supplementary Figure 5B).

Given that the legume *SOC1* genes have similar patterns of expression, we examined their promoters with the aim of identifying conserved regulatory elements. Short regions of high conservation were identified within the various legume promoters (Supplementary Figure 6A). Examination of these conserved regions revealed potential transcription factor-binding sites. Notably, there were multiple MADS-box transcription factor-binding sites (Supplementary Figure 6B). In *Arabidopsis*, the *SOC1* promoter is bound by the MADS-box proteins, FLC, SVP, and SOC1 itself (Searle et al., 2006; Immink et al., 2012; Tao et al., 2012; Mateos et al., 2015). Potential SPL, WRKY, and bHLH transcription factor-binding sites were also found in the legume *SOC1* promoter regions (Supplementary Figure 6B).

### *MtSOC1b* and *MtSOC1c* Up-regulation in Response to Inductive Conditions Requires *MtFTa1*

**FIGURE 4:**
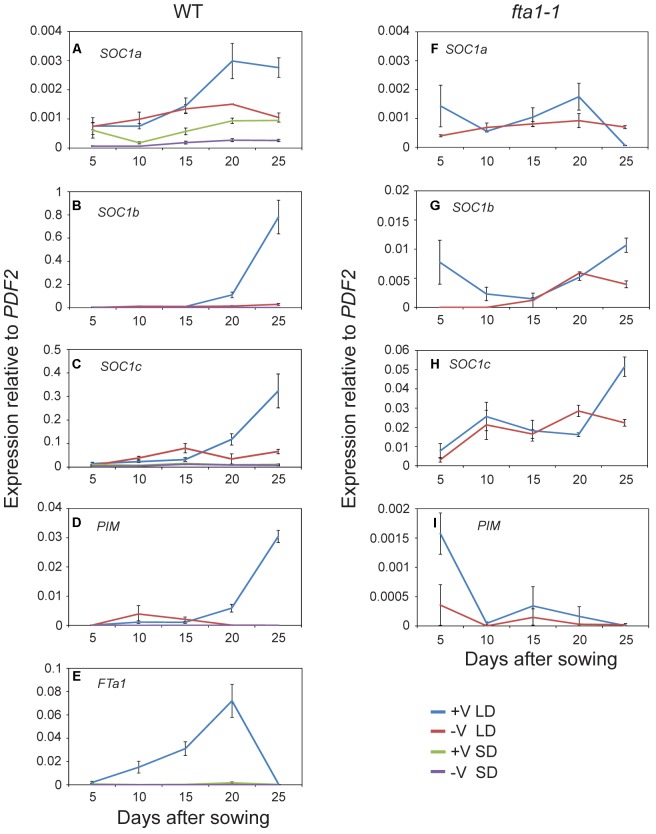
Time course of *MtSOC1* expression during development. **(A–E)** Expression of indicated genes in wild-type R108 and **(F–I)** in *fta1-1* (R108 background). Wild-type plants were grown under LD and SD photoperiod, with and without vernalization. *fta1-1* plants were grown under LD alone, with and without vernalization. All treatments were sown into soil at the same time. Aerial tissues of plants excluding cotyledons were harvested for days 5–15 and apical nodes only for days 20 and 25. Data are the mean ± SE of 2–4 biological replicates and transcripts were normalized to *PDF2*. All samples were harvested at ZT 2.

Given the potential role of the *MtSOC1* genes in flowering time, we examined their expression during development under different environmental conditions. As *Medicago* flowering is promoted by both vernalization and LD photoperiods, wild-type plants (accession R108) were grown with and without vernalization (± V) under LD (16 h light:8 h dark) and SD (8 h light:16 dark) photoperiods. Germinated seeds of all treatments were sown into soil at the same time. Gene expression was examined in aerial tissues (excluding cotyledons) for samples collected at days 5–15 after sowing, and in apical nodes at days 20 and 25 (**Figure 4** and statistical analysis of this data is shown in Supplementary Figure 7). *MtSOC1a* was the lowest expressed *MtSOC1* gene throughout the experiment, but was expressed at the highest levels in the vernalized LD samples at days 20 and 25 (**Figure 4A**). *MtSOC1b* was the most strongly expressed gene under inductive conditions in WT and was induced by day 20 (**Figure 4B**), with high expression by day 25. In contrast, *MtSOC1b* was relatively weakly expressed under SD (**Figure 4B**). However, it did show higher expression under LD photoperiod even without vernalization, compared to vernalized and non-vernalized SD treatments (Supplementary Figure 8). *MtSOC1c* behaved in a similar fashion to *MtSOC1b*, being strongly up-regulated under inductive conditions in WT, although to a lesser extent (**Figure 4C**).

The role of *MtFTa1* in the induction of the *MtSOC1* genes was also addressed in this experiment, by examining the expression of *MtSOC1* in the late-flowering and vernalization insensitive *fta1-1* (R108) mutant background (Laurie et al., 2011). In *fta1-1* mutants grown under LD, both *MtSOC1b* and *MtSOC1c* were expressed at similarly low levels irrespective of vernalization, and expression was dramatically attenuated compared to vernalized wild-type plants (for *MtSOC1b* and *MtSOC1c* approximately 100-fold, **Figures 4G,H**), suggesting both these genes are common targets of the vernalization pathway and are downstream of *MtFTa1.*

Expression of the FMI gene, *MtPIM* (Benlloch et al., 2006), was examined in all conditions tested (**Figures 4D,I**). Similar to *MtSOC1* genes in WT plants grown under inductive conditions, *MtPIM* was up-regulated in apical nodes by day 20, thereby heralding the commitment to flowering, and confirming the timing of *MtSOC1* up-regulation is consistent with these genes having a role in the floral transition at the apex. Furthermore, only vernalized LD conditions resulted in a gradual up-regulation of *MtFTa1* in WT plants (**Figure 4E**), which is necessary to rapidly promote flowering under favorable environmental conditions (Laurie et al., 2011; Jaudal et al., 2013). In wild-type, *MtFTa1* induction is concomitant with *MtSOC1b* and *MtSOC1c* activation in apical nodes, in both vernalization and LD photoperiod conditions, over the duration of this experiment.

### Regulation of *MtSOC1* by Photoperiod Shift

**FIGURE 5:**
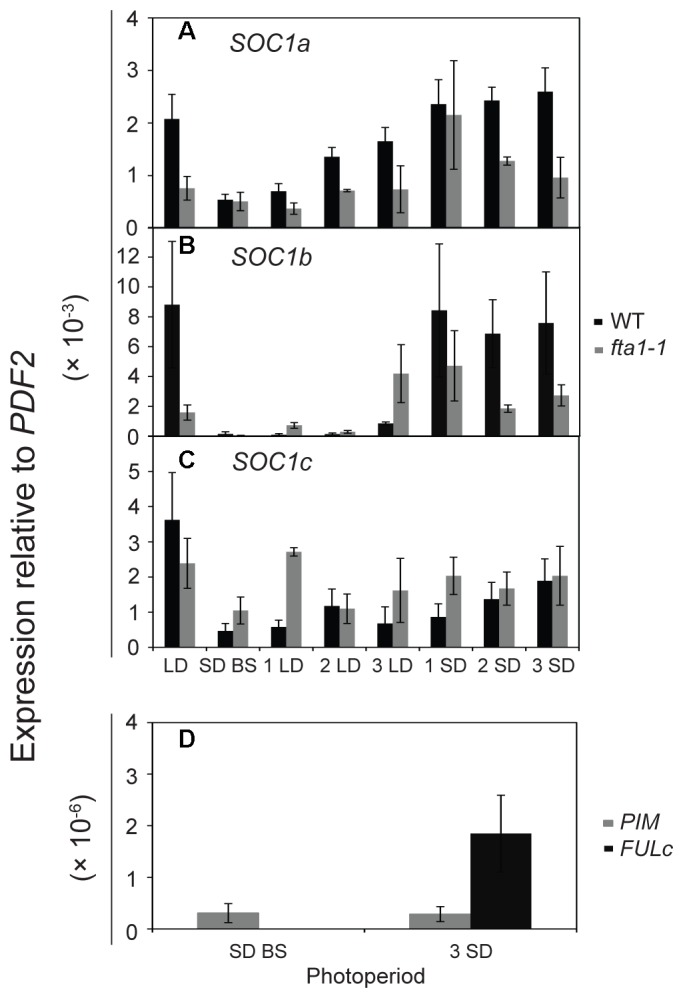
Regulation of *MtSOC1* in apical nodes by changes in photoperiod. Expression of **(A)** *MtSOC1a*, **(B)** *MtSOC1b*, **(C)** *MtSOC1c*, and (**D**) *MtPIM* and *MtFULc*. Vernalized WT (*c.v*. R108, black) and *fta1-1* (R108, gray) plants were grown under SD photoperiod until the monofoliate leaf had appeared (∼6 days). Plants were sampled [SD-BS refers to SD sample collected before shifting (BS) into LDs] and then transferred to LD photoperiod for sampling for 3 days (1 LD, 2 LD, and 3 LD). The remaining plants were then returned to SD for 3 days and sampled each day (1 SD, 2 SD, and 3 SD). LD control plants were grown permanently under LD and harvested at the conclusion of the experiment. Samples are the mean ± SE of 3 biological replicates and transcripts were normalized to *PDF2*. All tissues were harvested at ZT 4 in both light regimes.

As the *MtSOC1* genes are up-regulated under inductive LD photoperiods (**Figure 4**, Supplementary Figure 7), we sought to examine in greater detail the effect of photoperiod on *MtSOC1* expression in apical nodes. Since exposure of SD-raised vernalized WT (accession R108) plants to just 3 LD is sufficient to commit *Medicago* to flowering (Laurie et al., 2011), we investigated whether *MtSOC1* is induced in the SAM after this period of exposure to LD photoperiod, and the role of *MtFTa1* in this activation (**Figure 5** and statistical analysis of this data is shown in Supplementary Figure 9). In plants permanently grown under LD, which were harvested at the conclusion of the experiment (the equivalent of 3 SD time point), *MtSOC1a* and *MtSOC1b* were expressed at higher levels in WT than *fta1-1* (**Figures 5A,B**). All three *MtSOC1* genes were relatively weakly expressed in SD before shift (SD BS) conditions (**Figures 5A–C**). Upon transfer of WT plants into LD, *MtSOC1a* and *MtSOC1b* were up-regulated and remained high when transferred back to SDs (**Figures 5A,B**). An increase in *MtSOC1a* and *MtSOC1b* expression was also evident when *fta1-1* mutant plants were shifted into LDs, and generally this increase was smaller than for WT plants (although none of the changes in expression were statistically significant; Supplementary Figure 9). Although *MtSOC1c* is expressed at higher levels in LD grown plants, compared with SD grown plants (**Figure 4C**), no statistically significant up-regulation in *MtSOC1c* expression was observed over the six days of this experiment (**Figure 5C**). To check the LD exposure was sufficient to induce markers of the floral transition, we examined the expression of the FMI genes, *MtPIM* and *MtFULc* (Benlloch et al., 2006; Jaudal et al., 2015), in WT of pre-shift SD samples, and in samples harvested at the conclusion of the experiment, which had seen both 3 LD and subsequently 3 SD (**Figure 5D**). *MtPIM* expression remained unchanged between these time points, while *MtFULc* was activated following return to SD, suggesting that *MtFULc* is likely activated before *MtPIM*.

Overall, our results indicated that LDs promote the expression of the three *MtSOC1* genes (**Figures 4**, **5**). However, the dynamics of this LD response varied between the *MtSOC1* genes, with *MtSOC1b* showing the largest increase in expression after exposure to three LDs, *MtSOC1a* showing a moderate increase and *MtSOC1c* showing little response (**Figure 5**). This rapid induction of *MtSOC1a* and *MtSOC1b* is not eliminated in the *fta1-1* mutant (**Figure 5**), likely due to the involvement of other LD-inducible *MtFT* genes, such as *MtFTb1*(Laurie et al., 2011). In contrast, the large increase in *MtSOC1a* and *MtSOC1b* expression is probably associated with floral induction (as indicated by the up-regulation of the FMI gene, *MtPIM*) and is not seen in the *fta1-1* mutants which flower late and are yet to express *MtPIM* (**Figure 4**). Consistent with this idea, *MtSOC1b* expression begins to increase at later in development in the *fta1-*1 mutant (Supplementary Figure 8).

### *Medicago soc1b* Mutants Do Not Flower Late

**FIGURE 6:**
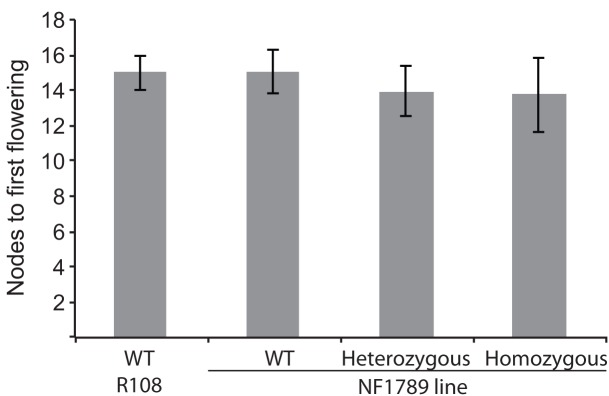
The *soc1b* mutant does not flower late. Flowering time of vernalized wild-type R108 and insertion mutant line NF1789 grown under LD photoperiod. Individual plants of a segregating NF1789 line were genotyped to determine if they were wild-type, heterozygous or homozygous mutants. Data are mean ± SE of 8 or more plants. There is no statistically significant difference between the flowering times of the lines.

To identify mutations within the *MtSOC1* genes, a reverse genetics approach was employed to screen for Tnt1 retrotransposon-tagged insertion mutants, sourced from the Noble Foundation collection (Tadege et al., 2008) by PCR. Two independent lines in the *c.v.* R108 background were found to have Tnt1 insertions within exon 7 of *MtSOC1b* (Supplementary Figure 10A). Homozygous plants for an insertion annotated as NF1789 were identified using PCR primers binding within the *MtSOC1b* gene and the Tnt1 insertion. No qRT-PCR products were detected using primers across the insertion site, indicating that the Tnt1 sequences prevent a correctly spliced mRNA from being produced (Supplementary Figure 10B). Homozygous NF1789 plants flowered at a similar time to wild-type (R108), when grown under inductive conditions (**Figure 6**). Moreover, a second independent insertion mutant, line NF9471 (also with a Tnt1 insertion within exon 7), also flowered at a similar time to wild-type (Supplementary Figure 10C).

## Discussion

While detailed knowledge of how flowering time is regulated exists for *Arabidopsis*, a member of the Brassica family, less is known about how flowering is regulated in other plant families. There are several differences between *Arabidopsis* and legumes relating to flowering time control, with evolutionary and genetic data indicating the likely involvement of legume-specific genes and mechanisms. For example, legumes lack a clear ortholog of the key *Arabidopsis* vernalization-responsive gene, *FLC* (Hecht et al., 2005) and the ability to respond to vernalization has likely evolved independently in legumes and other plant families (Bouché et al., 2017). Orthologs of the key gene in the *Arabidopsis* photoperiodic flowering pathway, *CONSTANS* (*CO*) do not function to control flowering of *Medicago* (Wong et al., 2014). Here, we set out to investigate the *SOC1* genes of *Medicago truncatula* and their role in the control of flowering time.

The *SOC1* gene plays an important role in *Arabidopsis* flowering by integrating multiple floral cues within the apex to promote flowering at the appropriate time. We have found that legumes contain two classes of *SOC1* genes, which we refer to as Group A and Group B *SOC1*s, as a result of a duplication of a region containing ∼20 genes. This duplication is present in diverse legumes and likely a result of the WGD that occurred during the early evolution of the papilionoid legumes about 58 million years ago (Bertioli et al., 2009). This WGD has been important in the evolution of various legume-specific traits allowing the duplicated genes to acquire new functions, while the other gene copy maintains its original function (Young and Bharti, 2012). In the case of the two groups of *SOC1* genes, they differ in their amino acid sequences and patterns of expression, indicating that they might have evolved distinct functions.

Despite the duplication that resulted in two groups of legume *SOC1*s and the more recent *Medicago*-specific tandem duplication, all three *MtSOC1* genes are able to promote the flowering of the *Arabidopsis soc1-2* mutant. Similar phenotypes have also been reported for the two Group A *GmSOC1* genes, with *GmGAL1/GmSOC1* (Glyma18g224500) being able to partially complement the late-flowering *soc1-1* (Ler) mutant, and causing early flowering when constitutively expressed in a wild-type *Arabidopsis* (Col) background (Zhong et al., 2012). *GmSOC1-like*, the other Group A member (Glyma09g266200), also resulted in early-flowering when heterologously expressed (Na et al., 2013). Similar to *Arabidopsis* and *GmSOC1* genes (Lee et al., 2000; Samach et al., 2000; Zhong et al., 2012; Na et al., 2013), *Medicago SOC1* genes are expressed in a range of tissues, though at differing levels. All three *MtSOC1* genes are expressed in apical node tissue, consistent with a potential role in control of flowering time, as reported for *GmSOC1* genes under inductive SD conditions (Na et al., 2013). *MtSOC1b* and *MtSOC1c* are also expressed at relatively high levels in stem tissue, indicating that they might also play a role in other aspects of *Medicago* development, such as internode elongation.

All three *MtSOC1* genes were up-regulated by inductive conditions of LD and vernalization, in a spatio-temporal manner concomitant with *MtFTa1* and *MtPIM* induction in leaves and shoot apices, respectively. *Medicago fta1-1* mutants do not respond to vernalization (Laurie et al., 2011), indicating that *MtFTa1* is an important and perhaps the sole direct target of the vernalization process. Consistent with this hypothesis, there are no clear differences between the expressions of the different *MtSOC1* genes in an *fta1-1* mutant compared to wild-type grown without vernalization. This suggests *MtSOC1* genes can be placed genetically downstream of *MtFTa1* in the vernalization pathway. Supporting this conclusion, studies on early-flowering *Medicago spring* mutants, which show LD-conditional ectopic *MtFTa1* expression, have higher levels of *MtSOC1a* expression in both leaves and apices, compared to wild-type (Jaudal et al., 2013). The requirement for *MtFTa1* for vernalization, as well as *MtSOC1* induction, has been further highlighted in *Medicago vrn2* mutants that flower early under LD photoperiod without vernalization(Jaudal et al., 2016). *vrn2-1* mutants show elevated levels of *MtFTa1* under LD photoperiod, accompanied by increased levels of all three *MtSOC1* genes in leaf tissue. *vrn2-1 fta1-1* double mutants flowered late like *fta1-1* single mutants, which was accompanied by a suppression of *MtSOC1* expression in the double mutant back to levels approaching wild-type (Jaudal et al., 2016). Taken together, these data from us and others clearly demonstrate that both *MtFTa1* and vernalization are essential for maximal *MtSOC1* induction, in response to favorable seasonal cues.

*MtSOC1* expression is also up-regulated by LD photoperiod in the absence of vernalization (therefore with low *MtFTa1* expression) (**Figure 4** and Supplementary Figure 7). We have previously shown that shifting *Medicago* plants from non-inductive SDs into inductive LDs for just 3 days, is sufficient to both up-regulate two functional *MtFT* genes, *MtFTa1* and *MtFTb1*, and to commit to flowering in plants that are subsequently returned back SD photoperiod (Laurie et al., 2011). *MtFTa1* and *MtFTb1* are both rapidly up-regulated in the leaves, while *MtFTa1* is also up-regulated in the shoot apex. The expression of *MtFTa1* and *MtFTb1* rapidly declines upon returning plants to SD photoperiod (Laurie et al., 2011). Here, we show that 3 days of *MtFTa1* expression is sufficient to induce the expression of *MtSOC1a* and *MtSOC1b* genes (**Figures 5A,B**). *MtSOC1a* and *MtSOC1b* expression also remains at higher levels when the plants are shifted back into SDs. Consistent with both *MtFTa* and *MtFTb* genes having a role in LD induction of flowering in legumes (Hecht et al., 2011; Laurie et al., 2011), this induction was reduced, but not eliminated, in the *fta1-1* mutant background. The rapid induction of *MtSOC1a* and *MtSOC1b* suggests that they might be early targets of *MtFT* genes. In *Arabidopsis*, *SOC1* is also rapidly up-regulated during the floral transition (Samach et al., 2000). The FMI gene, *MtFULc* (Jaudal et al., 2015), was also induced six days after plants were first transferred into LDs, however, the *Medicago* ortholog of *AP1*, *MtPIM*, remained unchanged. *MtSOC1c* also did not show strong up-regulation upon exposure to LD in the photoperiod shift experiment, whereas in the time course experiment both *MtSOC1c* and *MtPIM* were up-regulated (**Figure 4**). This raises the possibility that these two genes could be more important slightly later during the *Medicago* floral transition. Following flowering, the expression of both *MtSOC1b* and *MtSOC1c* remained at high levels in floral nodes, indicating a potential role in floral development.

Overall, the results of this study indicate that *Medicago SOC1* genes act downstream of the *FT* genes and likely act redundantly to promote flowering. Obtaining double and triple mutants’ combinations for the three *SOC1* genes will be needed to determine their importance in flowering and potentially other aspects of *Medicago* development.

## Author Contributions

JF, REL, and RM designed the experiments. JF, RHL, REL, and JLW performed the experiments. KM and JW identified the *Medicago soc1b* mutants. JF and RM wrote and edited the manuscript.

## Conflict of Interest Statement

The authors declare that the research was conducted in the absence of any commercial or financial relationships that could be construed as a potential conflict of interest.

## Footnotes

1 http://plantgrn.noble.org/AGED/SearchVisual.jsp#
