# Supplementary material for: Medicago truncatula SOC1 Genes Are Up-regulated by Environmental Cues That Promote Flowering
Source: Front Plant Sci. 2018 Apr 27;9:496. doi: 10.3389/fpls.2018.00496 (PMC5934494; doi:10.3389/fpls.2018.00496)
Supplement: Supplementary file 1 [file Presentation_1.PDF]

Supplementary Material

Medicago truncatula SOC1 genes are up-regulated by environmental cues that promote flowering

Jared B. Fudge, Robyn H. Lee, Rebecca E. Laurie, Kirankumar S. Mysore, Jiangqi Wen, James L. Weller and Richard C. Macknight*

*** Correspondence:** Corresponding Author: Richard.Macknight@otago.ac.nz

1 Supplementary Figures and Tables

1.1 Supplementary Figures

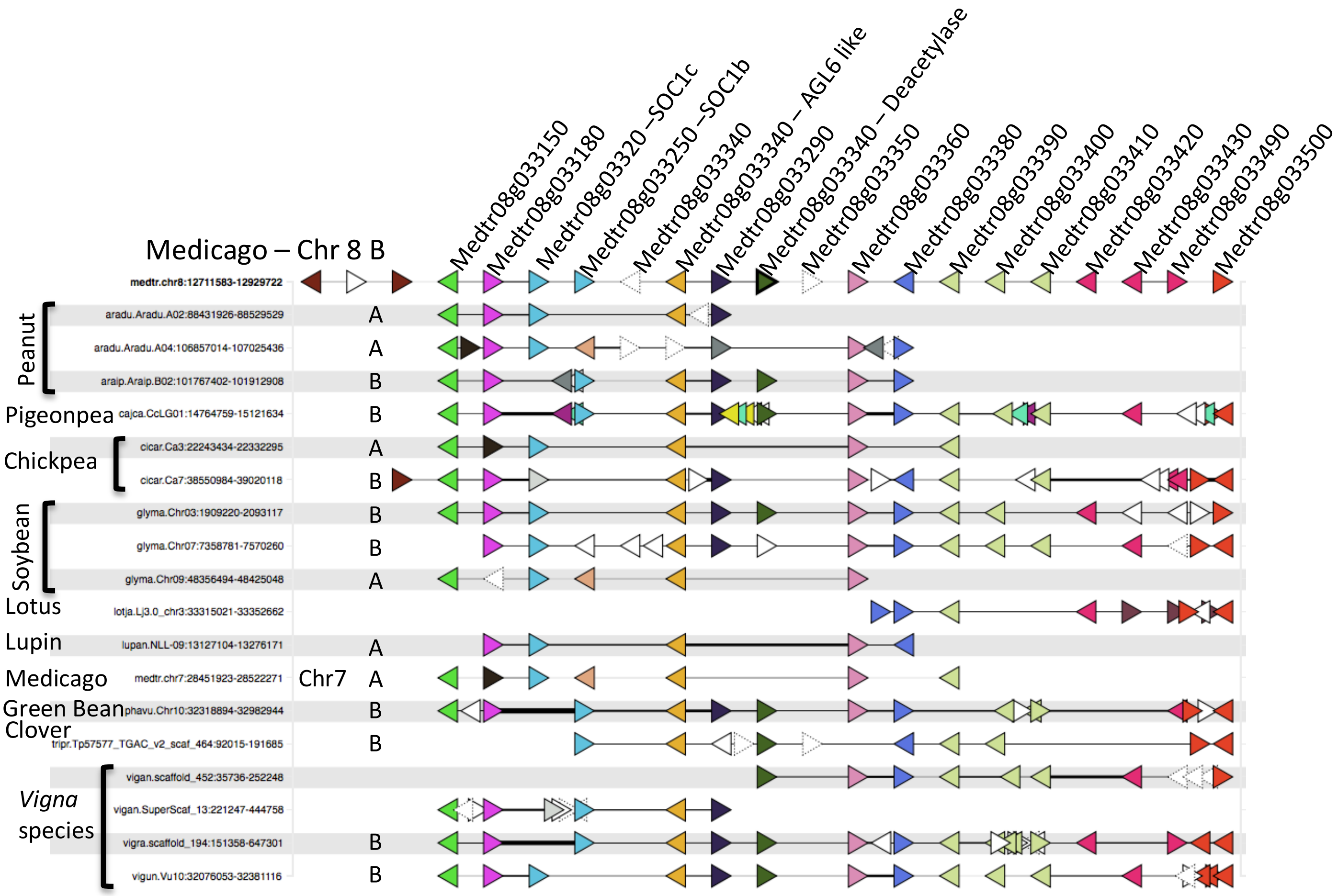

Supplementary Figure 1. Genomic structure of loci adjacent to Group A and B *SOC1* genes in various legume species.

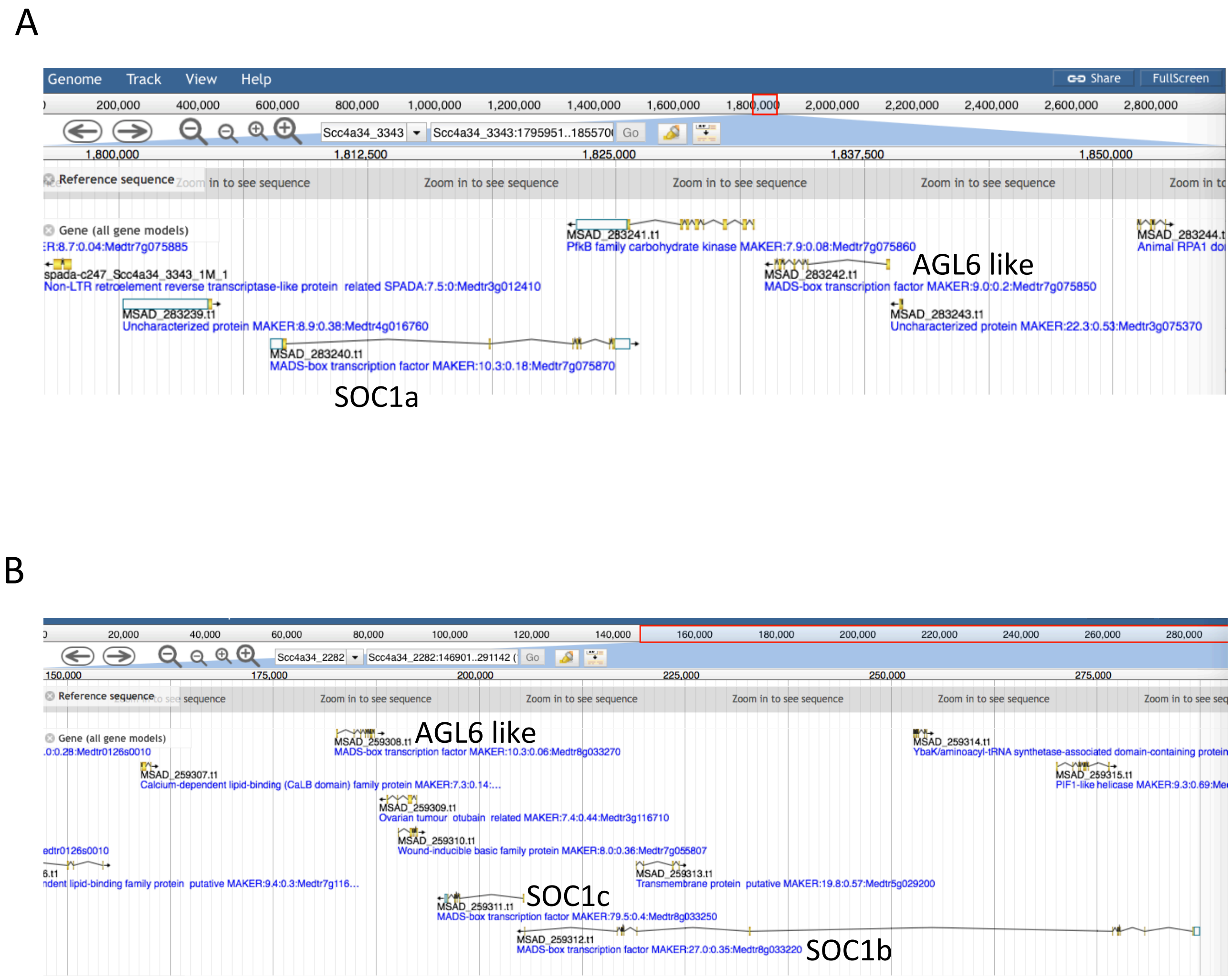

Supplementary Figure 2. *Medicago sativa* contains *SOC1a*, *SOC1b* and *SOC1c* genes. Chromosomal regions containing the *SOC1a* gene (**A**) and the duplicated *SOC1b* and *c* genes (**B**). The location of the *AGL6-like* genes is also shown. Pictures are screen shots from the *Medicago sativa* Genome browser available at www.alfalfatoolbox.org.

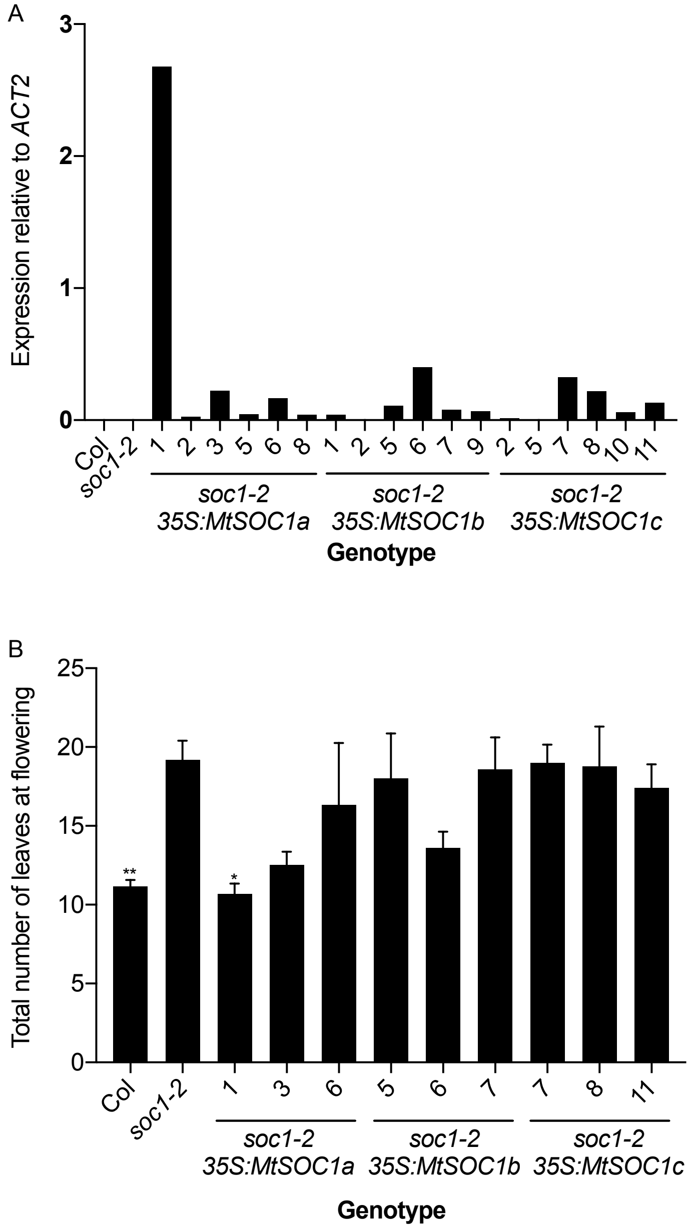

Supplementary Figure 3. Characterization of selected *soc1-2* (Col) transgenic lines harbouring *35S:MtSOC1* cassettes. (A) *MtSOC1* transgene expression levels in six independent lines. RNA was isolated from pooled LD-grown whole seedlings and used to quantify *MtSOC1* expression by qRT-PCR. Three lines showing the highest expression levels per construct were subsequently scored for their flowering time phenotypes in (**B**). Expression was normalized to *AtACTIN2* **(B)** Flowering time phenotypes of selected T_3 transgenic lines harbouring *35S:MtSOC1* constructs in the *soc1-2* (Col) mutant background. Plants were grown under LD photoperiod and the total number of leaves was scored at flowering. Data are mean ± SE and statistically significant differences between means of genotypes compared to *soc1-2* were determined by one-way ANOVA with Dunnett's test. Asterisks, where annotated, denote P values. * P ≤ 0.05, ** P ≤ 0.01.

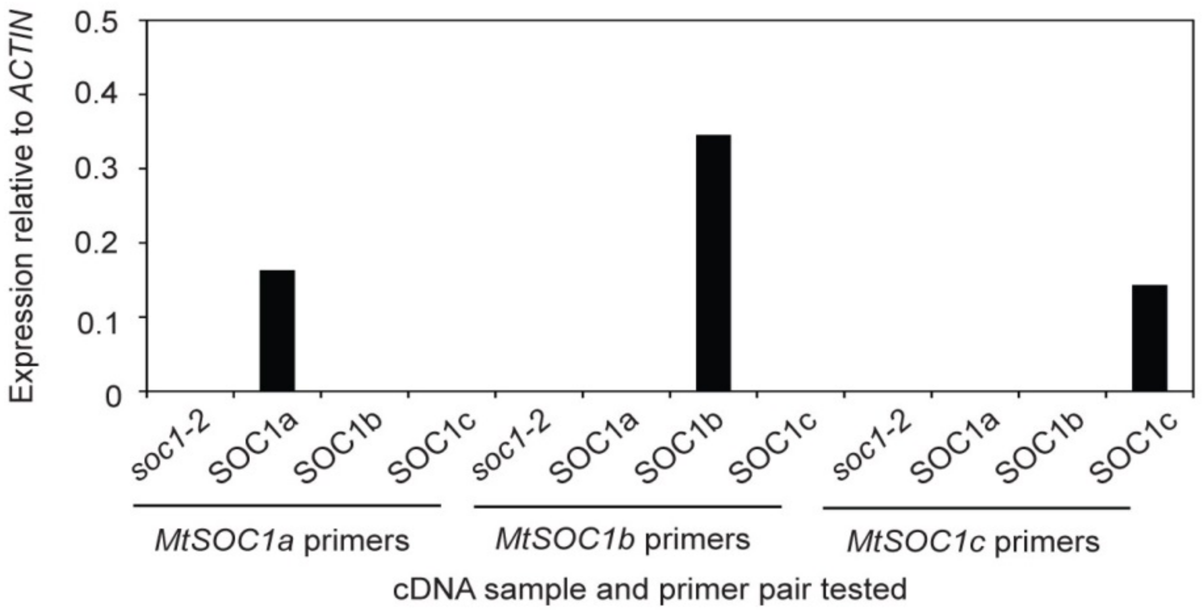

Supplementary Figure 4. Primers for qRT-PCR are specific to each *MtSOC1* gene. cDNA samples from transgenic Arabidopsis lines expressing *35S:MtSOC1* sequences in the *soc1-2* (Col) background were used as template for amplification by qRT-PCR using primers designed to amplify each *MtSOC1* gene, in the presence of the other *MtSOC1* genes and absence of wild-type *AtSOC1*. Expression was normalised to *AtACTIN2*.

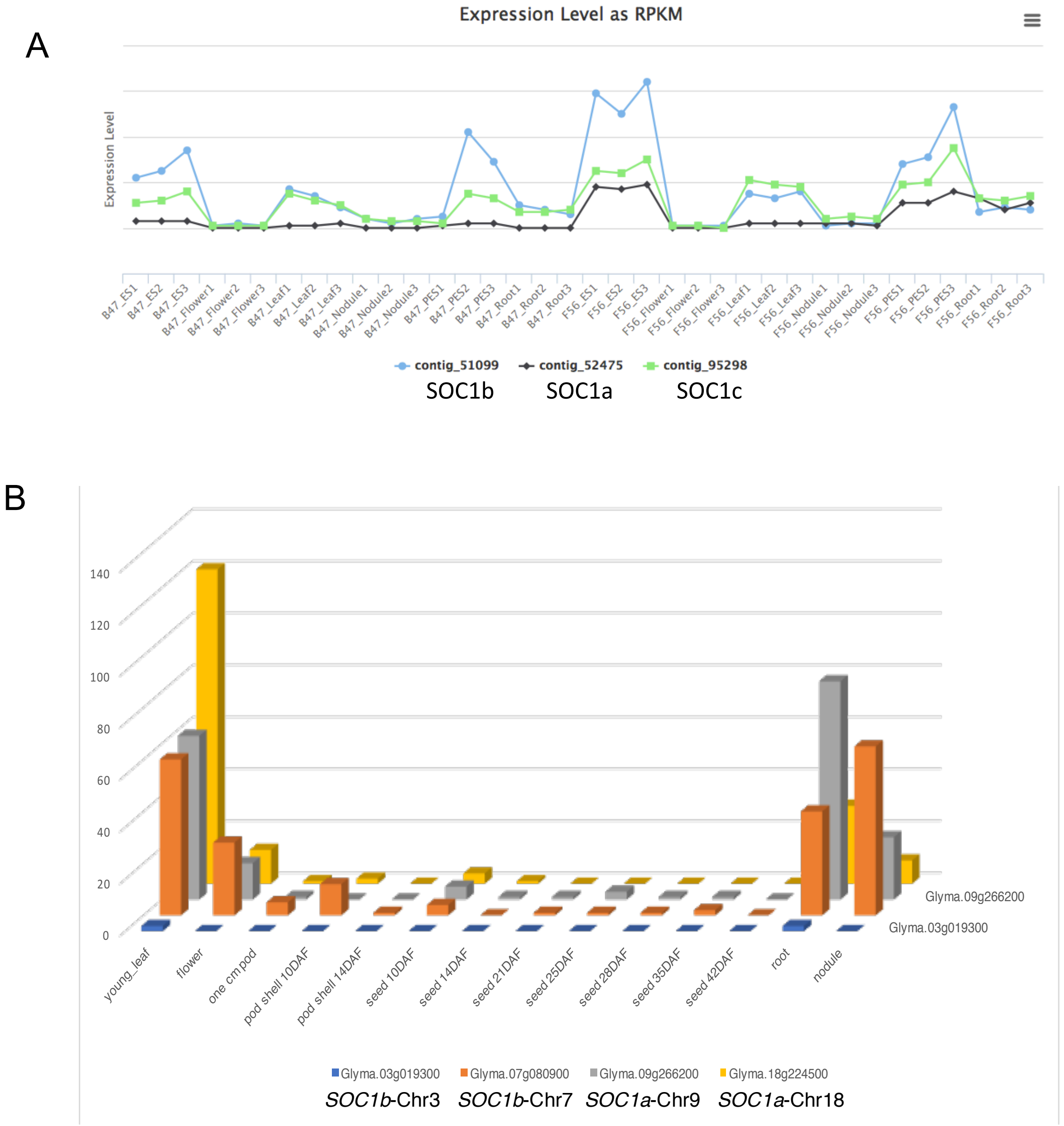

Supplementary Figure 5. Expression of group A and B *SOC1* genes from other legumes. (A) *Medicago sativa* contigs representing *SOC1a*, *SOC1b* and *SOC1c* transcripts. Samples are: B47_ES1-3 (Elongating Stem Internodes), B47_Flower1-3 (Developing Flowers), B47_Leaf1-3 (Mature Leaf), B47_Nodule1 (Nitrogen Fixing Nodules), B47_PES1-3 (Post-Elongating Stem Internodes), B47_Root1-3 (Whole Root), F56_ES1-3 (Elongating Stem Internodes), F56_Flower1-3 (Developing Flowers), F56_Leaf1-3 (Mature Leaf), F56_Nodule1-3 (Nitrogen Fixing Nodules), F56_PES1-3 (Post-Elongating Stem Internodes), and F56_Root1-3 (Whole Root). 1-3 refers to the number of biological replicates. Data was obtained from The Alfalfa Gene Index and Expression Atlas Database, available at http://plantgrn.noble.org/AGED/SearchVisual.jsp#. **(B)** Soybean RNA SEQ Atlas data for the four soybean *SOC1* genes. Data is from https://soybase.org/.

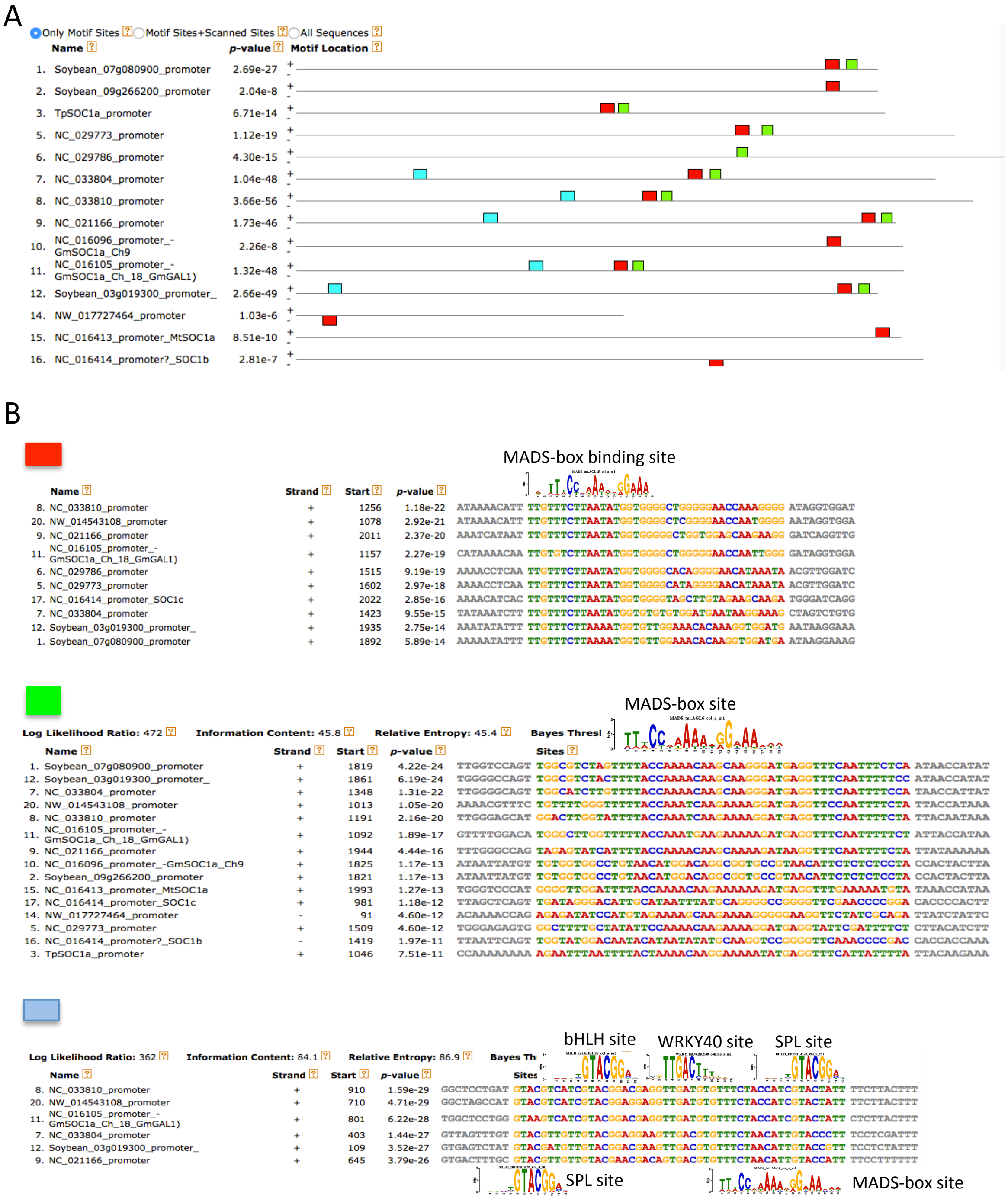

Supplementary Figure 6 Promoter analysis of legume *SOC1* genes. 5′ regions (defined as ~2 kb of sequence upstream of the start codon) were analyzed using the online program MEME (Multiple Em for Motif Elicitation) available at http://meme-suite.org/tools/meme. The Tomtom program (http://meme-suite.org/tools/tomtom) was used to identify transcription factor binding sites within the identified conserved motifs based on known *Arabidopsis* transcription factor binding sites. (**A**) Relative locations of identified motifs as shown by color codes in panel B. (**B**) Consensus sequences and alignments of motifs found to be enriched in legume *SOC1* 5′ regions.

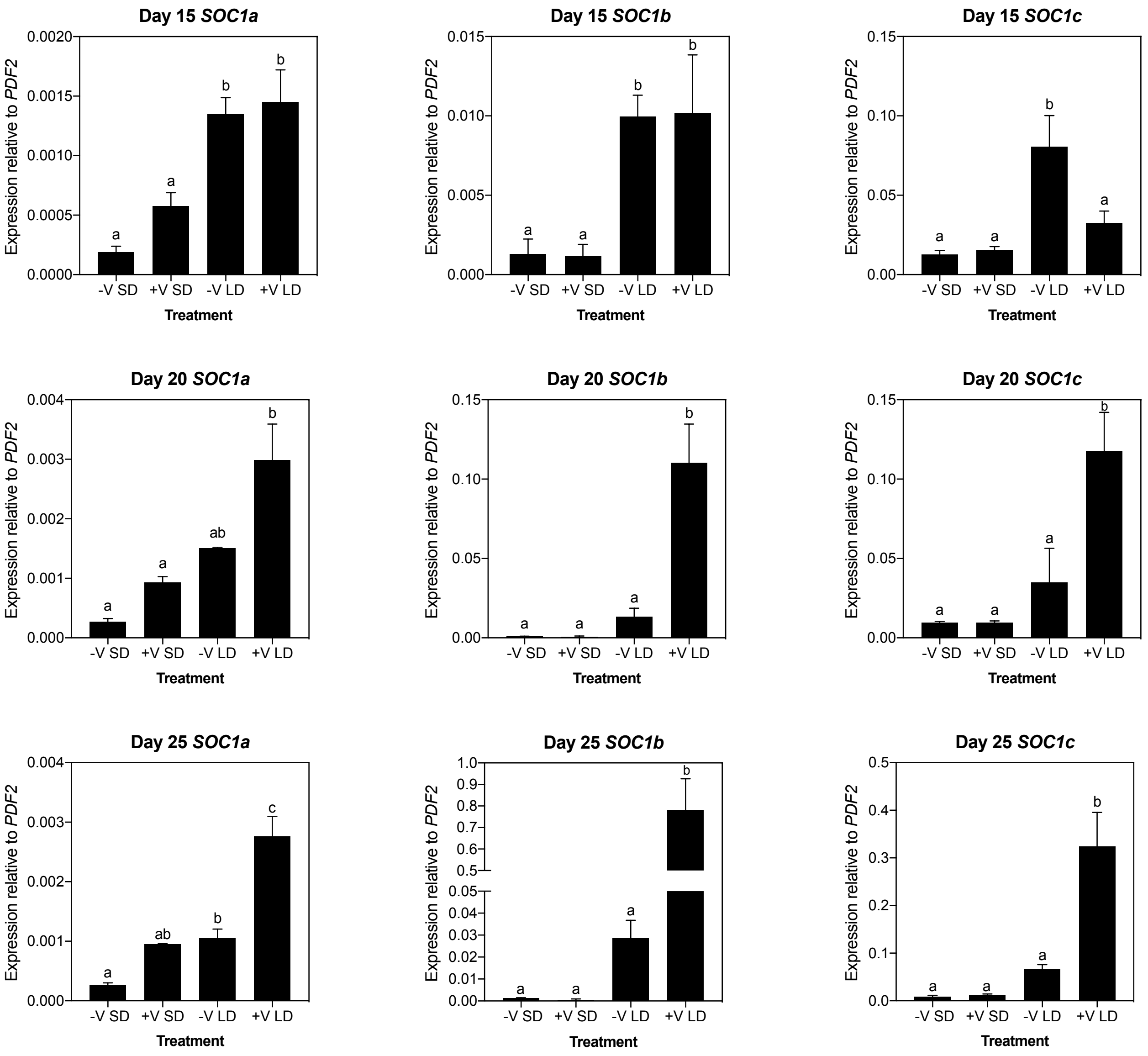

Supplemental Figure 7. Effect of vernalization and photoperiod on *MtSOC1* expression during development. Plants were grown and harvested as described in Figure 4. Data were re-plotted to compare the effects of vernalization and photoperiod treatments on *MtSOC1* expression at days 15-25 after germination. Statistically significant differences between means of each treatment were determined by one-way ANOVA with Tukey's test for multiple comparisons. Data are the mean ± SE of 2-4 biological replicates and data were normalized to *PDF2*. Different letters indicate statistically significant differences ($P < 0.05$) between means for each treatment.

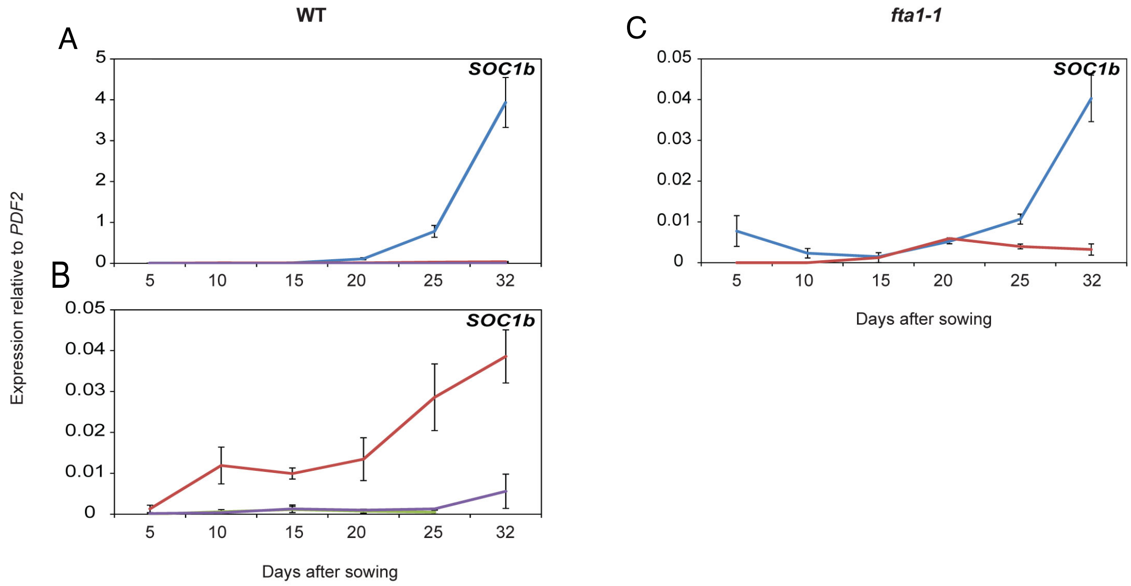

Supplementary Figure 8. *MtSOC1b* expression during plant development. Data from Figure 4B in WT R108 (reproduced here in panel **A** were re-plotted here in panel **B** in the absence of data points from vernalized LD conditions, and the y axis re-scaled. **(C)** Shows *MtSOC1b* begins to increase in *fta1-1* mutants after 32 days.

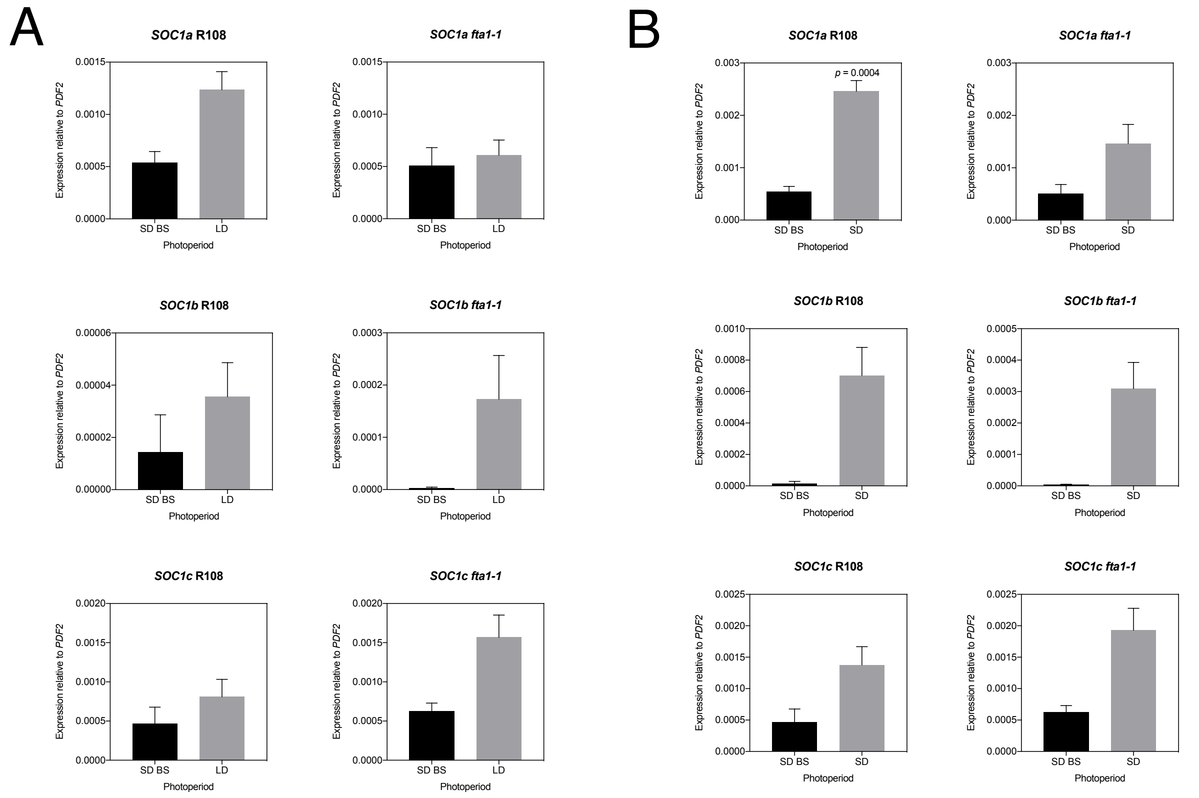

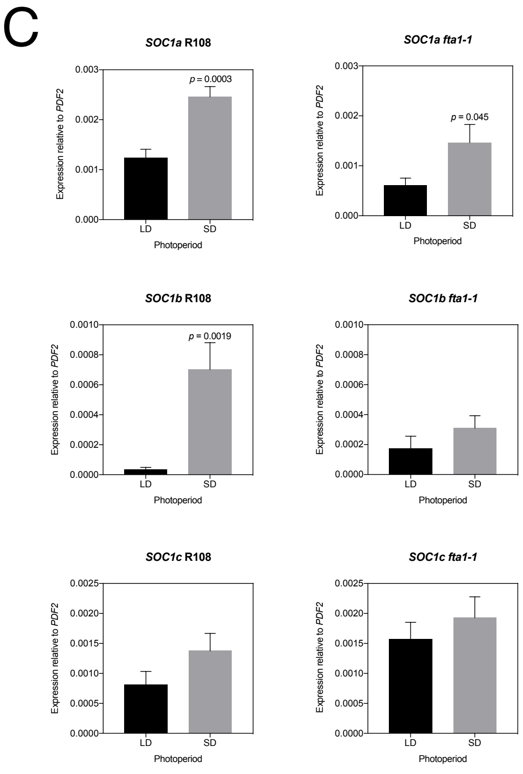

Supplemental Figure 9. Statistical analysis of data from photoperiod shift experiment. Data **from Figure 5 were re-plotted to compare the effects** of photoperiod treatments on *MtSOC1* expression. Statistically significant differences between means of each treatment were determined by two-tailed t-tests **(A)** SD BS data was compared with combined data from LD 1, LD 2 and LD 3. (**B**) SD BS data was compared with combined data from LD 1, LD 2 and LD 3. Statistically significant differences between means for each treatment are annotated for those with P values less than 0.05.

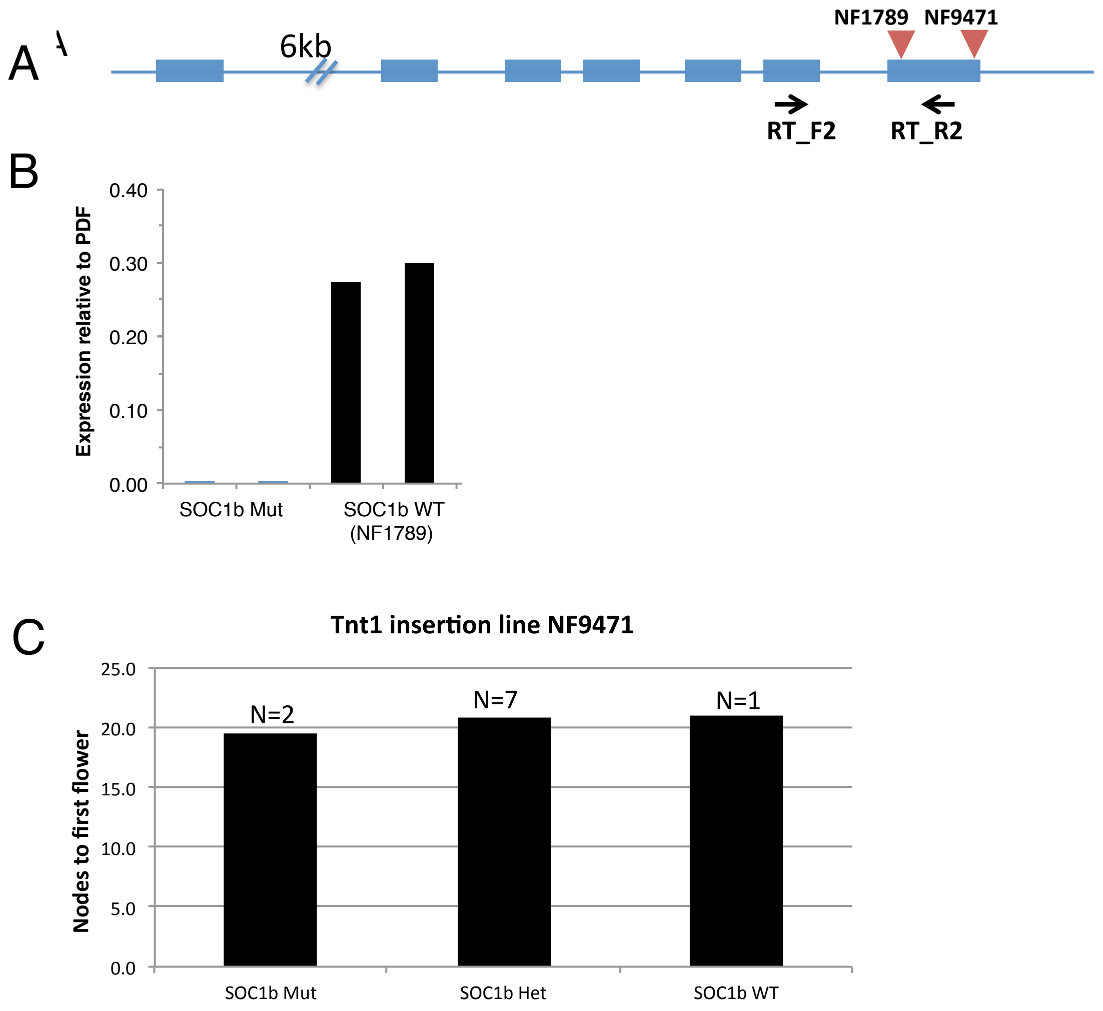

Supplementary Figure 10. Characterization of additional Tnt1 insertions in *MtSOC1b*. (A) Schematic of the *MtSOC1b* locus with insertion sites of two independent Tnt1 retrotransposon lines (indicated by triangles) shown. **(B)** *MtSOC1b* expression in wild-type (R108) and homozygous NF1789 mutants. Expression is relative to *PDF2*. **(C)** Flowering time of line NF9471 plants (heterozygous and homozygous individuals) and wild-type c.v. R108. Plants were vernalized and grown under LD photoperiod.

Supplementary Table 1. List of oligonucleotide primers used in this study.

Gene	Mt3.5 or GenBank ID	Sequence of oligonucleotide (5′ to 3′)	Source
MtPDF2 F	TC107161	GTGTTTTGCTTCCGCCGTT	Kakar *et al.* 2008
MtPDF2 R		CCAAATCTTGCTCCCTCA TCTG	
MtSOC1a F	Medtr7g075860	GCATTTGAGCTATCTGTT	This study
MtSOC1a R		GTAGCGTTCAATTGTGT	
MtSOC1b F	Medtr8g033250	GCTGAAAATGTCAGGCTCTCT	This study
MtSOC1b R		GACAATGAACAATTCAGTCTCC	
MtSOC1c F	Medtr8g033220	CTGAAAATGCCATGCTCGCA	This study
MtSOC1c R		AGACCAATGAACAATTCAGTTTC	
MtFTa1 F	Medtr7g084970	CTAGCAGTAGGAATCCACTAGC	Laurie *et al.* 2011
MtFTa1 R		GTGAATCAGGATCCACCATA	
MtPIM F	Medtr8g066260	ACGCAGAAACCAAGTCA	Laurie *et al.* 2011
MtPIM R		TGGAAGTTGCTGCTGTAG	
MtFULc F	Medtr7g016630	CTT GAC ACT TCG CTA AAG CGA AT	Laurie *et al.* 2011
MtFULc R		GCA TCT TTG TGG ATG TTC ACT C	
AtACTIN2 F	NM_112764.3	CGCTCTTTCTTTCCAAGCTCAT	Lee *et al*. 2013
AtACTIN2 R		TCCTGCAAATCCAGCCTTC	
MtSOC1b_F	Genotyping	ATGTCAGGCTCTCTGAGAAG	This study
MtSOC1b_R	Genotyping	GCATCAATACAGACATAGG	This study
Tnt1_F1	Genotyping	TCCTTGTTGGATTGGTAGCCAACTT TGTTG	Tadege *et al.* 2008
Tnt1_R	Genotyping	CAGTGAACGAGCAGAACCTGTG	Tadege *et al.* 2008

References for supplementary material

Kakar K, Wandrey M, Czechowski T, Gaertner T, Scheible W-R, Stitt M, Torres-Jerez I, Xiao Y, Redman J, Wu H, Cheung F, Town C, Udvardi M (2008) A community resource for high-throughput quantitative RT-PCR analysis of transcription factor gene expression in *Medicago truncatula*. *Plant Methods* **4**: doi:10.1186/1746-4811-4-18

Karimi M, Inzé D, Depicker A (2002) GATEWAY™ vectors for *Agrobacterium*-mediated plant transformation. *Trends in Plant Science* **7:** 193-195 doi: 10.1016/s1360-1385(02)02251-3.

Laurie R, Diwadkar P, Jaudal M, Zhang L, Hecht V, Wen J, Tadege M, Mysore K, Putterill J, Weller J, Macknight R (2011) The Medicago *FLOWERING LOCUS T* homolog, *MtFTa1*, is a key regulator of flowering time. *Plant Physiology* **156**: 2207-2224 DOI: https://doi.org/10.1104/pp.111.180182

Lee R, Baldwin S, Kenel F, McCallum J, Macknight R (2013) *FLOWERING LOCUS T* genes control onion bulb formation and flowering. *Nature Communications* **4**: doi:10.1038/ncomms3884

Tadege M, Wen J, He J, Tu H, Kwak Y, Eschstruth A, Cayrel A, Endre G, Zhao PX, Chabaud M, Ratet P, Mysore KS (2008) Large-scale insertional mutagenesis using the Tnt1retrotransposon in the model legume Medicago truncatula. Plant J. 54: 335-47. doi: 10.1111/j.1365-313 X.2008.03418.x
